# Balance between Health Risks and Benefits for Outdoor Workers Exposed to Solar Radiation: An Overview on the Role of Near Infrared Radiation Alone and in Combination with Other Solar Spectral Bands

**DOI:** 10.3390/ijerph17041357

**Published:** 2020-02-20

**Authors:** Carlo Grandi, Maria Concetta D’Ovidio

**Affiliations:** Department of Occupational and Environmental Medicine, Epidemiology and Hygiene, Italian Workers’ Compensation Authority (INAIL), 00078 Monte Porzio Catone, Rome, Italy; m.dovidio@inail.it

**Keywords:** solar radiation, near infrared, outdoor workers, immune response, adverse effects, preventive and protective measures, health surveillance

## Abstract

Near infrared or infrared A (IRA) accounts for over 40% of the solar spectrum (SS) and is able to reach subcutaneous tissue as well as the retina. Outdoor workers are occupationally exposed to solar radiation (SR), but the level of exposure may differ widely depending on the job performed, time spent outdoors, latitude, altitude, season, personal protection, etc. Until now, risk assessment and management for outdoor workers has focused on the prevention of both acute and long-term effects on the eye and the skin due to solar ultraviolet radiation (UVR) with little consideration of the other components of the SS (a possible exception is represented by visible radiation with reference to the eye). A growing body of evidence coming from in vitro studies indicates that IRA is involved in cellular reactive oxygen species (ROS) production and may interfere with the respiratory chain in the mitochondria. Moreover, it can modulate gene expression and some metabolic pathways. The biological action of IRA is only partly attributable to a thermal mechanism, should it be also involved in photochemical ones. The cellular and molecular pathways affected by IRA are partly similar and partly different with respect to those involved in the case of visible ultraviolet A (UVA) and ultraviolet B (UVB) radiation. Consequently, the net effect of the SS is very difficult to predict at different levels of the biological organization, making more difficult the final balance of health risk and benefits (for the skin, eye, immune system, blood pressure, etc.) in a given exposure situation. Moreover, few in vivo studies and no epidemiological data are presently available in this regard. Investigating this topic may contribute to better defining the individual exposome. More practically, it is expected to bring benefits to the risk assessment and management for outdoor workers exposed to SS, contributing to: (1) better definition of the individual profiles of susceptibility, (2) more focused preventive and protective measures, (3) better implementation of the health surveillance and (4) a more effective information and training.

## 1. Introduction

The spectral composition of SR at the ground includes over 6% of UVR, 38–39% of visible radiation and about 55% of infrared (IR) radiation. Most of UVR (over 90%) is represented by UVA (315–400 nm) and, on average, 10% is UVB (280–315 nm). Ultraviolet C (UVC) (100–280 nm), which is a part of SR, is completely blocked by the ozone layer and by the oxygen contained in the upper layers of the atmosphere [1,2,3]. Visible radiation ranges from 380–400 to 700 nm, while IR, which constitutes about half of the whole SR, covers the spectral range from 700 nm to 1,000,000 nm and extends between far visible radiation (red) and the upper end of the radiofrequency spectrum. It is subdivided into three bands: near IR (or IRA, 700–1400 nm), mid IR (infrared B or IRB, 1400–3000 nm) and far IR (infrared C or IRC, 3000–1,000,000 nm) [4,5]. Each object whose temperature is higher than −273.15 °C (the absolute zero) emits IR and the emission spectrum is a function of the fourth power of the absolute temperature (Stefan-Boltzmann law). The most important natural source of IR is the sun and other localized sources are represented by geological ones (e.g., lava flows) as well as natural occurring fires. A large number of man-made sources are recognized. However, most of them emit in the IRB and IRC range, given the relatively low surface temperature. Only few (for instance incandescent and molten metals and glass) have a significant component of the emission spectrum lying in the IRA range. The largest natural source of IRA is the sun, accounting for over 40% of the total SR reaching the soil. The rest of IR, corresponding to 10–15% of the total SR, is represented by IRB (up to 2500 nm). The amount of IRA varies as a function of time of day and season. However, during the diurnal cycle IRA variations are less marked than those of SR as a whole (see for instance [6]). Cloudiness may affect the amount of IRA at the ground, as water absorbs in the IRA range, but IRA changes are less considered with respect to other SR components (e.g., UVB and UVA). The interest in solar IRA with regard to the potential health effects on exposed people is increasing. Apart its relative abundance in SR, several other reasons are involved, discussed extensively in the rest of the paper.

Outdoor workers are a significant component of the workforce in all countries. Unlike indoor workers, they are exposed to severe microclimatic conditions and to SR. Following an estimation of the European Agency for Safety and Health at Work about 14.5 million EU workers are exposed to SR for at least 75% of their working time (90% of them are men) [7]. This is likely to be an underestimation of the real number of outdoor workers. Moreover, a number of formally indoor workers are exposed to UVA and IRA components of SR through window glasses. Outdoor workers are likely to be at higher risk for both acute (erythema) and long-term (for instance skin cancer and cataract) effects of SR, but a reliable quantification of the risk is not still possible. The role of each component of SR in combination with the other ones is not yet clear and concerns regarding the balance between adverse and potential beneficial effects of SR are very difficult to address in the case of outdoor workers.

The aim of this paper is to focus these concerns, starting from biological/health effects of IRA that are suggested from research conducted to date. The possible interactions with other spectral bands of SR (especially UVB and UVA) and the role of IRA in determining the balance between adverse and beneficial effects of exposure to SR in outdoor workers are discussed, with a sub-focus on the immune system. Finally, preventive and protective measures, including proper health surveillance, are stressed for outdoor workers, showing research needs and perspectives. Before discussing concerns regarding solar IRA, it is useful to summarize biological and health effects due to SR, largely attributed to UVR.

## 2. Exposure to SR: Adverse and Beneficial Effects

**Figure 1:**
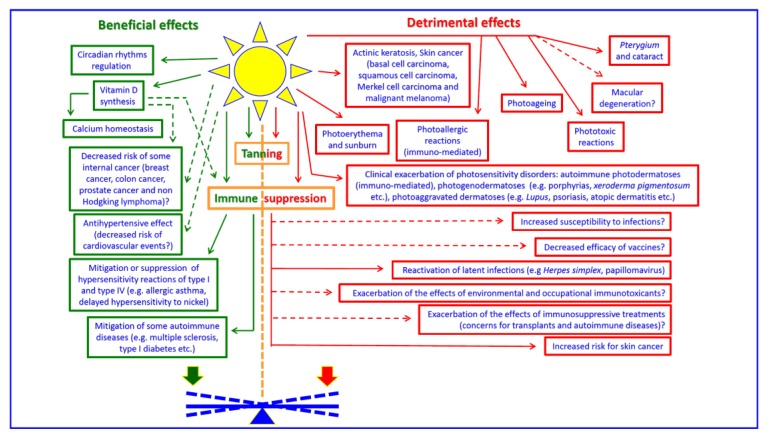
An overview of the health effects due to SR (Solar Radiation). Question marks and dashed arrows indicate effects not yet established or not yet fully established. Most of SR health effects are induced by UVB (UltraViolet B) and UVA (UltraViolet A), but for other ones visible radiation may be directly involved (e.g., circadian regulation, a subset of phototoxic and photoallergic reactions and macular degeneration). The balance between potential beneficial and detrimental effects in a given situation of exposure and for a given individual is often very difficult to address.

Figure 1 is a summary picture of the effects currently attributed to SR exposure (developed based on relevant literature, e.g., [2,4,8,9,10,11,12,13,14]). Some key features emerging from the analysis of the Figure 1 are the following:

1. Exposure to SR may induce effects on the skin and eyes, both acute and long-term. Among the long-term effects, the most well-known are melanoma and non-melanoma skin cancers. Merkel cell carcinoma also deserves a brief mention. This is a primary cutaneous neuroendocrine carcinoma characterized by a high malignancy (it represents the second cause of skin cancer death after cutaneous malignant melanoma) and with a rapidly increasing incidence in the last decades. Merkel cell carcinoma is associated with human Merkel cell polyomavirus; recognised risk factors include fair phototypes, impaired immune function and exposure to UVR.
2. Effects are mostly attributed to UVB and UVA components of SR.
3. Skin and eye represent the biological targets of SR, but exposure may induce effects (for instance the vitamin D synthesis) having great importance for several body tissues or the body as a whole.
4. Beneficial or potential beneficial effects are partly dependent and partly independent from vitamin D synthesis. In the latter case, they are due to SR itself or are mediated by factors other than vitamin D.
5. Some effects (e.g., tanning) are at the same time a marker of exposure to potentially harmful levels of SR and an adaptive response (beneficial).
6. Immune suppression due to solar UVA and UVB is an acute effect that, if repeated over time, may result in potential beneficial or adverse effects (some of them not still proven), depending on complex interplays between the level of exposure, the individual health status and the co-exposures to a number of physical, chemical and/or biological agents with immunomodulatory action. Immune effects will be discussed later in the paper, in a frame including the SR as a whole with concerns related to solar IRA.

Currently, much of the debate on the balance between adverse and beneficial effects of exposure to SR is focused on the role of vitamin D (see for instance [13,15,16,17,18,19,20]. This is due, among others, to the following reasons: (1) vitamin D has been shown to modulate several biological functions, playing a pleiotropic role in the body (well beyond that of a simple calcium metabolism regulator), (2) vitamin D synthesis or intake may be reduced for different reasons (e.g., geographical, cultural, insufficient exposure to SR, improper diet, etc.), (3) vitamin D insufficiency or deficiency may occur in population subgroups, (4) there is not yet a full agreement on vitamin D levels regarded as optimal, suboptimal, insufficient or deficient. The claim that insufficient or deficient levels of vitamin D may involve even large amounts of people in both developed and developing countries remains controversial. In any case, there is no doubt that a number of questions related to vitamin D deserve attention and further research is required. However, in our opinion it is equally important to view the balance of adverse vs. beneficial effects of SR with a full inclusion of non- (or partly non-) vitamin D related effects [9,21,22,23], having regard to the role played (or potentially played) by the rest of the SS in the context of ambient temperature and other ambient variables or exposures. In this regard, solar IRA may be of concern for the reasons extensively argued in the following.

## 3. IRA and Target Tissues

As for other optical radiation, IRA interacts with skin and eye tissues. The energy incident on the skin is partly reflected and partly transmitted through the tissue. The transmitted energy is partly absorbed by tissue chromophores and partly scattered in the tissue volume. In the IR range, water is the main absorber in biological tissue given its abundance and absorption spectrum, but some biomolecules or molecular complexes may absorb selective IR wavelengths (such as Cytochrome C Oxidase-CCO- in the mitochondria, see later). IRA penetrates more deeply into the skin than the rest of optical radiation and up to 17% of the incident IRA (at 1000 nm wavelength) may hit the subcutaneous tissue [24]. Consequently, many cell types may be exposed to IRA: keratinocytes, Langerhans cells, melanocytes, fibroblasts, endothelial cells of dermal vessels, adipocytes, etc.

**Figure 2:**
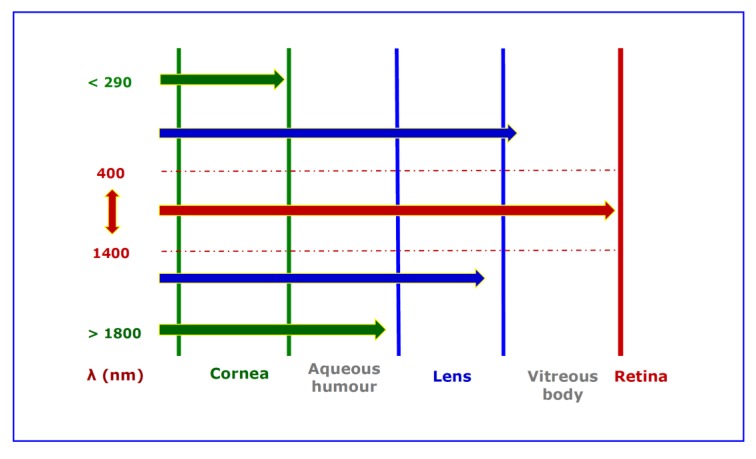
A schematic picture showing the ability of different spectral bands of optical radiation to penetrate eye. It is evident that only visible radiation and IRA (InfraRed A) may reach retinal tissue in physiological conditions.

The eye is highly transparent to visible radiation and, to a lesser extent, to IRA. Therefore, IRA may hit the retina [4,5] (Figure 2). 

## 4. Biological/Health Effects of IR

### 4.1. IR as a Whole

IR absorbed by tissue chromophores like water is converted into heat. Deep tissues may be heated when IR warms the superficial tissues (skin and cornea) and heat is then transferred in depth by conduction. The biological effects of heat largely depend on the extent of temperature increase above physiological temperature as well as on exposure duration [4,25,26]. Normal temperature is about 37 °C in the eye tissues (with the exception of the cornea) and varies physiologically from about 27 °C to 33 °C in the skin [27]. Consequently, skin has a higher margin of temperature oscillation than the eye, according to its extension and barrier function, but when temperature exceeds 39 °C pathological changes may occur. If tissue temperature approaches 41 °C heating effects take place but are still reversible. The critical temperature range is 43–45 °C for irreversible tissue damage, especially if such elevated temperatures are maintained for a prolonged time. At the cellular level heating induces defence responses, mostly focused on the Heat-shock proteins (Hsp), acting as chaperones [28]. Hsp are induced in response to a variety of stimuli (including heat and oxidative stress) and have multiple actions at cellular level. They include facilitation of the correct protein folding, involvement in the protein intracellular trafficking, involvement in the processes of removal of damaged macromolecules, participation to a variety of intracellular signalling pathways. The role of Hsp is not merely homeostatic, but also adaptive.

Depending on the attained temperature and the duration of heating, health effects may occur. In the IR spectrum, acute effects include skin and corneal burns. In the IRA range, an intense exposure may also induce thermal damage to the retina. A strong heating of the lens may result in acute cataracts, but less intense heating over prolonged exposures (month to years) may also induce cataracts. In the skin the only known chronic effect of heating is the so-called erythema ab igne [29,30], a reticular erythematous and hyperpigmented dermatosis described for repeated and prolonged exposure of a body district (usually legs, hands or face) to a localized source of heating (e.g., incandescent bodies, stoves, heating pads, hot water bottles, etc.). Skin areas with erythema ab igne are at higher risk to develop a squamous cell carcinoma. Heat burns or erythema ab igne are not reported for occupational or environmental exposure to SR, even for long durations of exposure.

### 4.2. IRA

Unlike what one may expect, IRA has not only thermal effects, but may display photobiological ones. Treatments of cell cultures with IRA lasers (selective wavelengths) or IRA emitted by artificial incoherent sources (with a broader spectrum) may affect cellular metabolism acting on the mitochondrial respiratory chain [31,32]. The net effect may be stimulatory or inhibitory, depending on irradiation parameters, but at least in part does not rely on temperature changes, indicating the occurrence of photochemical mechanisms. IRA, as well as the red part of the visible spectrum, has been used in phototherapy for many years. It is administered as coherent (laser) or incoherent (various types of lamps) radiation, exploiting the photobiostimulatory effects to treat a lot of conditions of the skin, subcutaneous tissue and other tissues by accelerating wound healing, reducing inflammation and, in general, stimulating metabolism (see for instance [33,34]). 

Photobiostimulation, in this context regarded as a beneficial effect, is not related or is not just related to tissue heating, which may be irrelevant or absent, as showed by experiments with a strict temperature control. It is largely attributed to the absorption of IRA photons of specific wavelengths by the mitochondrial CCO, causing its dissociation from the inhibitory nitric oxide (NO) and increasing oxygen consumption and ATP synthesis [35,36]. However, a full agreement in this regard does not yet exist [37]. The biochemical cascade triggered by IRA photons absorption leads to ROS production, activation of NF-kB (Nuclear Factor kappa-light-chain-enhancer of activated B cell) pathway, modulation of gene expression and, ultimately, cell proliferation, tissue repair and remodelling (including extracellular matrix synthesis). Experiments were often conducted using fibroblasts, which seem to be more sensible to IRA than keratinocytes and other skin cell types.

**Figure 3:**
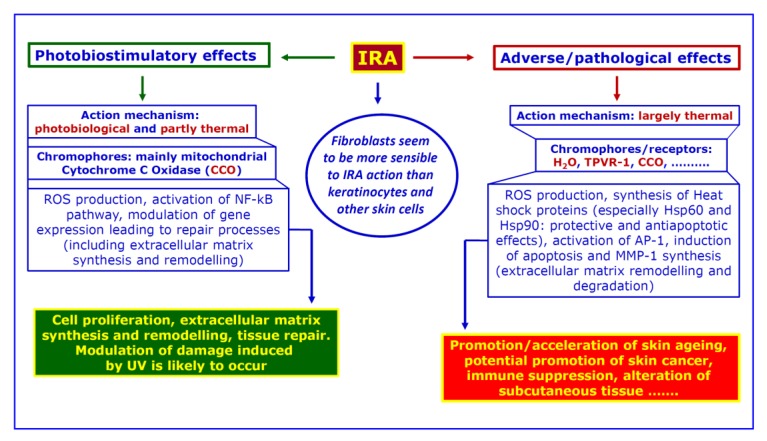
A simplified overview of the biological mechanisms underlying both photobiostimulatory and adverse/pathological effects due to IRA (InfraRed A) exposure, with indication of the possible outcomes. The prevalence of one type of effects over the other one is dependent on spectral composition, total dose, dose-rate, biological target and, possibly, other unknown factors.

Depending on dose rate (irradiance, in Watt per square meter-W/m^2^) and/or total dose administered in a given time (radiant exposure, in Joule per square meter-J/m^2^), the photobiological effects may be flanked by thermal ones, which may prevail in a number of exposure situations and could result in adverse or pathological consequences [31]. Unlike the case of biostimulation, water represents the main cellular chromophore when cell and tissue heating occur, but Transient Potential Vanilloid Receptor 1 (TPVR-1), a membrane protein acting as a heat sensor, may play a role in this regard. In the case of adverse effects, a higher ROS production (due to mitochondrial activity and/or to heat itself) and temperature changes lead to Hsp synthesis, stimulation of Hsp responsive elements (HRE), activation of Activator Protein 1 (AP-1) pathway, and the triggering of apoptosis and synthesis of Matrix MetalloProteinase-1 (MMP-1). This, in turn, may result in acute damage to the skin, but long-lasting effects may also occur. They include the acceleration of skin aging and, possibly, the facilitation of carcinogenic process (see for instance [24,31,38]). Stem cells depletion, extracellular matrix degradation and remodelling, survival of aberrant cell clones, and immune suppression are all mechanisms potentially involved in long-term outcomes. What is stated above is summarized in Figure 3.

The analysis of transcriptome in human primary fibroblasts revealed that IRA exposure (under a strict temperature control of cell cultures) may regulate the expression of about 600 genes, including genes related to extracellular matrix, calcium homeostasis, stress signalling and apoptosis, with a different pattern than that induced by UVR. IRA transcriptome was largely triggered by mitochondrial ROS [39]. Current data tend to support a biphasic response of the skin (but theoretically of other tissues) to IRA exposure, depending on wavelength/s and other irradiation parameters, notably dose and dose rate [6,31]. The role of each parameter is not fully determined. For instance, although some spectral subranges seem to be more effective in inducing a given biological response, a complete action spectrum of IRA-related biological effects is not available. 

The occurrence of a biphasic response of body tissues or the body as a whole to agents like ambient temperature is well known, but it seems to be a general rule. For instance, a number of metals are essential at low or very low dosage but toxic at higher doses. Exposure to ionizing radiation or toxic chemicals is damaging at medium-high doses but may induce an adaptive response at low doses. The phenomenon by which an agent is stimulatory (then potentially beneficial) at low doses and hazardous at higher doses is known as “hormesis” or “adaptive response.” Hormesis was shown to occur at all levels of the biological organization (cell, tissue or the whole body), for a variety of effects, both acute or long-lasting (including carcinogenesis), as well as in relation to physiological agents (see for instance [40,41,42]). The adaptive response may result in increased resilience of tissues and the whole body to environmental and physiological stresses. 

Moving back to IRA, evidence seems to indicate photobiostimulatory and potentially beneficial effects when the total dose administered is less than 10 joule per square centimeter (J/cm^2^), while adverse effects arise for doses higher than 120 J/cm^2^ [31]. In the dose range between 10 and 120 J/cm^2^ experimental evidence is less informative, but it can be argued that as the dose increases photobiostimulation progressively drops and photoinhibitory/adverse effects begin to appear. In the range 10–120 J/cm^2^ or in subsets of this range an overlapping of stimulatory/inhibitory effects could exist and the prevalence of one type over the other one is likely to depend on the biological effect concerned, biological target, dose-rate and possibly other variables. The contribution of dose rate (irradiance) in determining the outcome is not so clear [43,44], although it is conceivable that at very low irradiances even high doses should not be harmful. On the opposite, high irradiances (100 milliWatt per square centimetre (mW/cm^2^) or more) may warm tissues and are likely to be hazardous though the total dose administered is low. For intermediate values of irradiance (e.g., tens of mW/cm^2^) the question is difficult to address on the basis of current data. One hour of exposure to SR in the tropics during summer solstice at noon corresponds to more than 72 J/cm^2^ of IRA dose, with a mean irradiance ranging from 20 to 35 mW/cm^2^ [6].

Based on what was previously stated, a few hours per day of exposure to SR may result in total IRA doses falling in the range of possible induction of adverse effects to skin, especially during spring and summer. However, given the wide variety of exposure durations among individuals of the general population as well as outdoor workers, the different seasons and latitudes, the variability of meteorological conditions and the great differences in personal protection attitudes, daily exposure to solar IRA is likely to span from a few tens to several hundred J/cm^2^. It is anticipated that this claim, jointly with concerns previously described and considerations reported in the following of this paper, does not currently allow a reliable balance for adverse vs. beneficial effects of exposure to solar IRA.

Eye effects due to IRA exposure are far less known in terms of photobiostimulation/photoinhibition, but a dual effect (i.e., stimulation at low doses/dose rates and inhibition at high doses/dose rates) is not excluded from occurring, for instance, in retinal cells, choroid tissue and lens cells.

## 5. Solar IRA and the Whole SS

Humans are exposed to the entire SS. Consequently, questions arise about the combined and simultaneous exposure to IRA, IRB, visible radiation and UVR (UVA and UVB). In particular, it should be important to state if additive/synergistic or, on the opposite, modulatory/antagonistic interactions occur. In terms of biological action mechanisms, it is recognized that IRA, visible radiation, UVA and UVB are all able to increase ROS production in skin cells, although at different rates and with non-overlapping mechanisms [6,31,45,46,47,48,49,50]. The same is true for photoreceptors with regard to visible radiation (being also part of the visual cycle) but might occur in photoreceptors themselves as well as in cells of other eye structures following exposure to IRA and UVR.

Regardless of the source of ROS, an intracellular ROS increase beyond the physiological ranges may cause oxidative stress and triggers intracellular signal cascade/s leading, for instance, to antioxidant response, synthesis of stress proteins, apoptosis, DNA repair and cell proliferation [51,52]. 

In this regard, it is speculated that solar spectral bands inducing ROS synthesis may act in an additive/synergistic way. This implies that the total amount of ROS produced in a given cell type in a certain time period is more likely to cause oxidative stress, i.e., a pro-oxidant redox balance in the cell, with potential deleterious outcomes (induction of apoptosis, higher mutation rate, a more intense promoting action on mutated clones, etc.). A synergistic interaction between UVA/UVB on one side and visible/IRA on the other side could thus result in a higher risk of long-term outcomes, such as skin cancer, skin aging, cataracts and macular degeneration. Despite the abundance of in vitro and in vivo data relating to UVA and UVB separately, little is available on combined exposure to UVA/UVB and other solar spectral bands. An exception is given by the observation of synergistic effects between UVA1 (340–400 nm) and visible radiation in inducing skin pigmentation and erythema in ten subjects with Fitzpatrick skin phototypes IV, V and VI [53]. 

The occurrence of additive/synergistic or modulatory/antagonistic interactions could be affected by the relative abundance of a given spectral band in the SS as a function of time of day, season, cloud cover, etc. For instance, it was suggested that the relatively less solar IRA in early morning after sunrise as well as in late afternoon before sunset might have a beneficial photobiomodulatory effect [6]. In early morning, this may result in preconditioning of skin cells to the following exposure to high levels of solar UVB and UVA, i.e., in an adaptive response to the UVR-induced damage, while in late afternoon a stimulation of repair activity would take place (including DNA repair).

The scarcity of experimental data does not allow for the drawing of firm conclusions in this regard.

## 6. Solar IRA and Ambient Temperature

Since IRA may affect a biological target photochemically, thermally or both, depending on irradiation parameters, a parallel question is to what extent the effects of exposure to solar IRA are modulated by ambient temperature. As previously mentioned, physiological skin temperature varies from about 27 °C to 33 °C, depending on ambient temperature and other factors (e.g., physical exercise), displaying circadian oscillations. It was stated that temperature increase in tissues leads to the synthesis of Hsp, whose molecular activity reduces thermal damage and mediates the adaptive response of the cell to heat. Heat was shown to perturb the redox balance in a cell, affecting ROS production and metabolism [31]. The net effect of cell heating depends on the extent and duration of the heating itself, spanning from an adaptive response to irreversible heat damage and cell death by apoptosis or necrosis. It was proposed that the adaptive response mediated by Hsp safeguards biomolecular functions and pathways, inhibiting apoptosis and promoting cell survival. However, the other side of the coin should be the survival of skin cells previously mutated, for instance by exposure to solar UVR or other environmental mutagens, enabling them to proceed along the carcinogenic pathway if replicative or other promoting stimuli occur [54]. Moreover, in human keratinocytes UVB and heat stress may act synergistically by fostering the survival of mutated cells, with potential implication regarding the skin carcinogenesis pathway [55]. It is recognized that Hsp are involved in DNA repair mechanisms [56], whose increased or decreased effectiveness is, as known, related to the risk of neoplastic transformation of the cell.

A role of skin temperature in modulating skin cancer risk was also suggested by some authors (e.g., [57]). In other words, heat might exert a co-carcinogenic action in certain conditions. This issue is not new, as the effectiveness of UVR in inducing skin cancer may be higher in hot environments as shown by experimental studies conducted decades ago [58,59,60], but the causal or con-causal role of heating in carcinogenesis (or in determining other long-term outcomes) is not clearly established and is still an object of debate. 

For instance, some authors (e.g., [61]) state that in experimental animals the skin carcinogenic effectiveness of UVR is 3–7% higher for each additional degree of temperature. The same authors [62] concluded that data on the incidence of non-melanoma skin cancer in 10 U.S. areas are significantly related not only to the environmental UVR levels but also to the mean values of maximum daily temperatures recorded during the summer period. The effectiveness of UVR should increase by about 2% for each degree of temperature rise (with a more marked effect for squamous cell carcinoma). On the other hand, other authors, e.g., [63], found that elevated ambient temperatures administered before UVB reduce the risk of UVB-induced skin cancer.

In any case, the possibility that modulatory effects on skin cancer risk may be exerted, in addition to elevated ambient temperatures, by spectral bands of SR like IRA raises serious concerns about exposure to SR in outdoor settings, especially if exposure lasts for a significant part of daytime and repeatedly over months or years. An increased production of ROS (having a potential mutagenic and cancer promoting effect) combined with Hsp-enhanced survival may lead to a persistent increase in skin cancer risk, and, possibly, to a faster photo-aging. This concern might be even more critical in view of the ongoing climate change (CC) [64]. CC is expected to change the overall exposure to SR, but the direction of this change is not predictable, especially at the local level, being affected by altered meteorological conditions and modification in life habits in response to CC itself [11,65]. In any case, CC on the one hand involves a slight but progressive increase in mean ambient temperature worldwide and on the other hand is responsible for an amplification of weather variability. This implies, for instance, more frequent, intense and lasting heatwaves. In terms of possible short- and long-term outcomes, an unchanged or even reduced exposure of target tissues to SR could be balanced by an increased overall exposure to elevated ambient temperatures.

## 7. Immune Effects 

As mentioned in chapter 2, UVR may exert an immunomodulatory action. As reported in human volunteers and experimental animals [66,67,68,69,70,71], alongside a stimulation of innate immunity UVR causes a suppression of the acquired immune response. Immune suppression was shown for both UVB (peak at 300 nm, with a dose-dependent saturation effect) and UVA (peak at 360–380 nm, with a bell-shaped dose-response relationship) and occurs acutely during and after skin irradiation. Experimental studies indicate that UVR-induced immune suppression is both local and systemic and may affect delayed type hypersensitivity reactions. Exposure to SR may reactivate latent viral infections (e.g., herpes simplex virus) and epidemiological studies suggest that SR exposure may impair to some extent the effectiveness of vaccines [72,73,74]. As shown in Figure 1, several potential implications of SR-induced immune suppression (some of them involving a net beneficial effect while others result in adverse outcomes) are not yet clearly highlighted by experimental and epidemiological studies. Moreover, it is suggested that UVR-induced immune suppression may alter skin microbiome [75]. Concerns are related to the role of immune suppression in promoting cancer, including skin cancer [76,77,78], since immune surveillance is crucial in recognizing and eliminating neoplastic cell clones, acting as the last barrier against neoplastic cell proliferation and invasion. Among potential immune modulatory factors, ambient temperature could have an important (and possibly underestimated) role. For instance, core body temperatures over 40 °C lead to a decline of immune function, but, excluding fever, the effect of different patterns of temperature increase on acute and/or long-term immune responses are not yet clear [79]. The final outcome/s could be affected in different ways as a function of the extent, duration and frequency of temperature increase. Moreover, a local or systemic temperature rise could have different implications. 

A number of Hsp (Hsp 90, Hsp 70, Hsp 60, Hsp 40 and Hsp 27) are involved in different features of the immune response, for instance modulating the patterns of several cytokines [80]. As different patterns of Hsp may be induced by heat and oxidative stress, a still unclear but potentially relevant link between temperature and ROS-producing stimuli on one side and immune response on the other side could exist. Hsp induction may facilitate survival and, potentially, neoplastic transformation of mutated cells and there is evidence that interfering with Hsp activity may promote carcinogenesis based on the immunomodulatory effect of these proteins. For instance, suppression of Hsp27 and Hsp70 accelerates DMBA (Dimethylbenz(a)anthracene)-induced skin carcinogenesis in C3H/HeN mice [81].

Moreover, an immunomodulatory effect of IRA regardless of heating is suggested. For instance, under rigorous temperature control it was shown that, compared to heating alone, in female C57BL mice IRA (700–1000 nm, 90 J/cm^2^) activates epidermal Langerhans cells in both epidermal sheets and draining lymph nodes [82]. 

It is important to remark that animal data, especially if sparse, are only suggestive of an effect possibly arising in humans. Metabolic and immunological differences do exist between rodents and humans and, although studies regarding skin cancer are conducted on hairless animals, rodent skin is normally haired and not pigmented. However, molecular and cellular mechanisms involved in oxidative stress response, DNA repair and carcinogenic process are fundamentally the same. In the case discussed here, experimental results obtained so far may be viewed as a warning for more extensive studies.

## 8. Outdoor Workers

A number of working activities are conducted outdoors. There is no shared definition of “outdoor worker,” but for the scopes of this paper an outdoor worker is regarded as a worker spending a significant part of the daily working time in conducting one or more jobs outdoors. Outdoor workers are exposed to additional risk factors compared to the generality of indoor ones, such as severe thermal conditions, SR, adverse meteorological events, some chemical, physical and biological pollutants, outdoor allergens, etc. [12,83,84,85]. Moreover, they may be exposed to the same pollutants found indoors, but with different exposure patterns. Outdoor exposures and related health effects may be exacerbated by CC, the last one representing an additional concern in occupational health [86,87]. Outdoor workers are electively exposed to SR, but the effective duration of exposure during a working shift or a working day may be highly variable [12] depending on the time effectively spent outdoor, type of job performed, environmental albedo, shading and personal protection. Even for activities implying a potentially strong exposure to SR, like those performed by sea workers (e.g., sea fishing), the effective individual exposure may vary considerably [88]. Some working activities carried out in indoor settings (e.g., driving a vehicle on the road) may cause exposure to significant levels of SR, but in this case UVB and partially UVA are filtered by the vehicle glasses. However, IRA is not generally filtered by conventional window glasses, including the ones in vehicles, and thus workers inside are potentially exposed to IRA (in addition to a fraction of the incident UVA).

As a sound definition of outdoor workers is lacking, any reliable quantification of their number is very difficult. An indicative (not exhaustive) list of outdoor working profiles and activities is reported in Table 1.

## 9. Safeguarding Outdoor Workers from SR: Concerns about IRA 

A particular directive on the protection of workers exposed to natural optical radiation does not yet exist in Europe. However, European regulation on occupational health and safety states that the employer has the duty to assess all occupational risks [89]. Consequently, risks arising from exposure of outdoor workers to SR have to be properly assessed and managed. In this regard, skin cancer prevention is of paramount importance, but eye diseases are equally relevant, especially cataracts and retinopathies.

Given the great differences in SR exposure among outdoor workers even in the same job sector, an individual exposure assessment may be useful. The exclusive use of available exposure metrics based on geographical/meteorological data and atmospheric transfer models (e.g., the UV index, see for instance [90]) should not provide the real individual exposure. Accordingly, site and personal measurements are needed to account for the exposure amount depending on body position and surrounding albedo. Since the most dangerous effects of SR are attributed to UVR, the exposure assessment is often restricted to solar UVA/UVB by using spectroradiometer methods and/or personal dosimeters [91]. Approaches to assess cumulative exposure to solar UVR were developed or are developing [92,93].

Presently, exposure to solar IR (solar IRA in particular) may only be assessed by spectroradiometer methods. As previously mentioned, variability of solar IRA over daytime, season or due to meteorological conditions is less studied compared to solar UVR. Nevertheless, a more detailed knowledge in this regard should be useful for a better assessment of outdoor workers’ exposure to the total SR and as a basis for a more targeted exposure assessment. 

The protection of people against SR is discussed in several papers and is the subject of articles, reports, guidelines and recommendations (examples are [94,95,96,97,98,99,100,101]). 

Some documents are addressed to the general public (occasionally with indications/specifications for outdoor workers), while others deal with occupational exposure. Indications are sometimes graded on the UV index values and include avoiding or reducing exposure during central hours of the day, seeking shade, rehydrating the body, wearing protective clothes, use of sunglasses and sunscreen. The discussion of these items is beyond the scope of this paper, but it is important to point out that these recommendations are enforced by considering the above-mentioned potential additive or synergistic interactions between UVR and other solar spectral bands (IRA in particular). In fact, in light of the concerns previously stated it is likely that IRA daily doses, dose rates or cumulative doses typical of many outdoor workers lead the potential adverse effects to overcome the potential beneficial ones. 

It is important to point out that, in general terms, the effective implementation of sun protective measures in different outdoor occupational settings depends critically on risk perception and attitudes toward sun protection, which are still inadequate and heterogeneous among outdoor workers [102,103]. Unlike other occupational risk factors, in the case of SR the “acceptance” of protective means by the worker is of paramount importance [104].

To protect the skin and eyes from IRA exposure, some concerns are to be emphasized. The shielding of IRA due to clothing is highly variable depending on texture, thickness, colour, etc., but it is much less studied compared to the UVR and garments are not usually tested in this regard. Moreover, large body areas may be uncovered, especially during summer, and, if covered, it is very important to comply with thermal comfort needs. It is conceivable that shielding of IRA by clothing is less effective than in the case of UVR, given the higher wavelengths involved. Sunglasses may completely block UVR but are not specifically designed and tested to shield IRA. As mentioned in chapter 8, current window glasses do not shield IRA as well. However, an effective filtering of all optical radiation but light is possible in perspective by the use of smart glasses. The question of sunscreen use deserves additional considerations. Commercially available sunscreens do not confer a specific protection against IRA as they do not contain IRA absorbers, but a partial shielding of solar IRA may occur due to scattering. Formulations containing topical antioxidants are regarded as a good solution by some authors (see for instance [38,105,106]), especially if associated with the oral intake of antioxidants by means of diet or supplements. In any case, a *complete* sunscreen is not yet available, although the need of a broad-spectrum protection against SR is claimed in a number of papers (e.g., [107,108,109]). 

EU regulation on occupational safety and health does not explicitly include health surveillance of workers exposed to SR. In other words, health surveillance is not a duty in the case of occupational exposure to SR per se, but it is only provided for workers occupationally exposed to artificial optical radiation [110]. However, outdoor workers exposed to SR are generally surveyed in relation to other occupational hazards, for which the health surveillance is mandatory. Moreover, health surveillance may be implemented on worker’s request, if the latter is regarded as pertinent (i.e., related to occupational hazards) by the occupational physician. If this is the case, health surveillance practices cannot ignore adverse health effects due to SR, and must include in their protocols items such as the assessment of individual susceptibility profiles (e.g., phototype, presence of photodermatosis, etc.), the detection of early signs of SR adverse effects, a targeted information delivered by the occupational physician, etc. Overall, job fitness assessment of the worker has to take also into account SR related health risks. Moreover, since the biological targets of SR are predominantly skin and eye health surveillance has to include a scrupulous dermatologic and/or ophthalmologic examination. Unlike the generality of risk factors targeting internal organs, this allows an early diagnosis of long-term adverse effects, including skin cancer.

It is very hard to split SR into its components for health surveillance purposes, but the occupational physicians has to be aware that UVR is not the only relevant one, given that IRA and visible radiation exert a biological action which, in the case of IRA, is not simply thermal. An occupational physician has to take into account the possibility of additive/synergistic effects in relation to the outcomes concerned as well as the potential modulatory role of ambient temperature. Moreover, it should be noted that the co-exposure to irritant, sensitizing, photosensitizing and/or immunomodulating agents may exacerbate the incidence and/or the severity of clinical features associated with some skin and eye effects of SR. Finally, co-exposure to skin carcinogens (e.g., Polycyclic Aromatic Hydrocarbons—PAH) may synergise with SR as a whole and, possibly, with ambient temperature.

The above indicates that in the health surveillance of outdoor workers exposed to SR an assessment of the immune profile is of great importance, since the presence of an atopic status or conditions of mild to severe immune impairment may worsen health outcomes associated to SR exposure. 

## 10. Discussion

The exposure to SR is a risk factor for outdoor workers and the overall impact on health of a large (and potentially increasing) number of subjects is likely to be relevant. The study of the potential adverse and beneficial effects of SR cannot ignore the role played by all SS components, including IRA [111]. In light of concerns and remarks reported in this paper, an attempt to forecast whether a given pattern of exposure to SR turns into a net beneficial or, conversely, a net adverse effect for a given individual in a given exposure situation (for instance an outdoor worker doing a particular job) is premature. However, some considerations seem to be pertinent.

Unlike the general public, outdoor workers are not likely to develop an insufficient or deficient vitamin D status. Moreover, SR exposure patterns of the generality of outdoor workers should confer them potential health benefits not mediated or only partly mediated by vitamin D. Among these benefits, blood pressure lowering, a (possible) reduced risk for some internal cancers, attenuation of clinical features of allergies, autoimmune or immune-mediated conditions (e.g., psoriasis, multiple sclerosis, type I diabetes, etc.) have to be mentioned. However, the same patterns of exposure to SR may increase the risk of adverse effects on skin and eye, especially the long-lasting ones. The individual risk of an outdoor worker may increase to a different extent if one or more of the followings features are present, although a direct quantification of the risk relating to specific outcomes is not feasible.

The risk is predicted to increase in fair phototypes (I and II) and for exposure to SR during leisure time and vacation. A greater risk is also given in the case of poor personal protection during spring and summer seasons, for pre-existing medical conditions involving mild to severe immune impairment (e.g., HIV infection, conditions requiring immunosuppressive and antineoplastic treatments, etc.) and if co-exposure of the skin to immunotoxic chemicals, skin chemical carcinogens (e.g., PAH) and infectious agents occurs. Finally, lifestyles may modulate the individual risk of SR-induced adverse effects: for instance, the risk is likely to increase because of smoking or artificial tanning practices. 

Protection of outdoor workers exposed to SR should ideally include the shielding of IRA, which is now difficult to achieve optimally. Solar IRA represents a reason, besides others, to continue to explore in depth the role of topical and systemic antioxidants, including those active in mithocondria [112]. Some questions remain open, for instance the antioxidants of choice and the relative amount of a given antioxidant in a given formulation. In the case of antioxidants and/or epigenetic modulators contained in food or oral supplements it is necessary to address the question of ensuring suitable concentrations of the compound/s in the target tissues, defining the optimal dosages and avoiding overintake. Another concern deals with the potential interactions with drugs. An additional and crucial concern related to the administration of some antioxidant compounds is their potential ability, at least in some conditions, to act as pro-oxidants, driving the carcinogenic process instead to prevent cells to follow the carcinogenic pathways [113,114]. More generally, an alteration of the redox balance of the cell (in both senses) may result into unexpected and potentially serious consequences for cells, tissues and even the whole body and this issue has to be fully explored. 

Epidemiological studies focusing on solar IRA are not yet available. However, epidemiological investigations of proper size, design and conduction should contribute to define IRA role (alone and in combination with other SS bands) for a given health outcome as well as to identify suitable biomarkers of exposure, effect and susceptibility. There is a scarcity of data regarding the effects of IRA on retinal/coroid tissue and lens. New studies making use of suitable animal models and IRA sources more similar to the SS (such as solar simulators) should be very useful in this regard. Moreover, it is of great importance to define action spectra of IRA-induced photobiological effects and to address concerns related to dose and dose-rate for a better prediction of the balance between adverse and potential beneficial effects of SR exposure. Another research need is to explore epigenetic changes in cells of skin and eye tissues due to exposure to IRA (alone and combined with the rest of SR): this would lead to a better understanding of the action mechanisms and, in perspective, should be useful for risk assessment purposes. Studies in this regard are still lacking or are very scarce [115]. Finally, it should be noted that, similarly to what occurs in many other research fields, the introduction of “omics” approaches in both in vitro and in vivo studies can no longer be postponed. Once combined with a proper management of “big data” they allow to test the overall response of a biological system to a certain pattern of exposure/s, matching results with exposure features.

It should be noted that the large amount of information collected by complying with the aforementioned research needs would contribute to define the individual exposome for outdoor workers [116,117,118].

## 11. Conclusions

**Figure 4:**
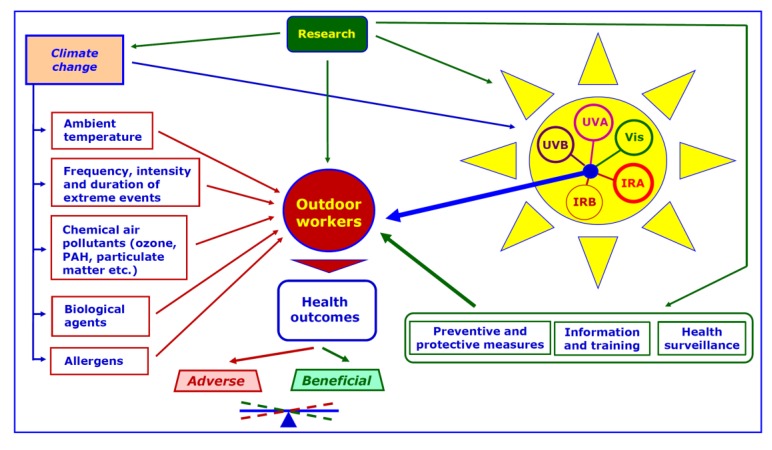
The balance between potential adverse and beneficial health outcomes for outdoor workers exposed to SR (Solar Radiation) depends on the whole SS (Solar Spectrum) and, in addition to the implementation of preventive and protective measures, is affected by a variety of environmental and occupational co-exposures which, in turn, may be modulated by CC (Climate Change). An extensive research is needed to address properly these concerns.

A more complete preventive and protective approach to outdoor workers exposed to SR requires to account for the whole SS as well as the complete set of potential health outcomes in outdoor scenarios characterized by different patterns of co-exposure to environmental and occupational physical, chemical and biological agents (Figure 4). 

Protective measures against solar UVR usually recommended for the general public and adapted to different types of outdoor activities confer a certain degree of protection against the whole SS. Outdoor workers may suffer from potential limitations to personal protection in different situations; examples are provided below:

1. Need to comply with thermal comfort (suitable choice of protective garments, i.e., garments protecting against UVR and SR in general but ensuring a proper transpiration and heat dispersion).
2. Need to comply with other personal protective devices (sometimes sunglasses should not be compatible with the necessity to protect eyes and face from splinters, vapours and particulate matter, thus requiring the choice of compatible personal protective devices).
3. A hot and dusty work environment could cause excessive discomfort and/or sweating following the use of certain sunscreen formulations (e.g., creams, thus requiring the choice of alternative formulations like sprays).

It is important to develop standardized approaches and methods to test new sunscreen formulations containing antioxidants or other compounds useful to absorb or scatter IRA and visible radiation. A similar claim is valid for garments and sunglasses. Preventive interventions have to include, among others, education to healthier lifestyles.

SR is ubiquitous and outdoor workers are an important component of the total workforce. In our opinion, the development of the research lines proposed above, albeit time-consuming and expensive, will have concrete effects on preventive and protective practices, resulting in a considerable reduction of morbidity and mortality of outdoor workers due to occupational and environmental related exposures. In particular, it should be possible to:

1. provide a better definition of the individual susceptibility profiles,
2. design more focused preventive and protective measures,
3. adopt better health surveillance practices,
4. deliver a more effective information and training, encouraging healthier lifestyles,
5. contribute to the individual and collective adaptation to environmental challenges, such as those related to CC.

## Figures and Tables

**Table 1:** A non-exhaustive list of outdoor working profiles and activities, distinguishing those with a prevalence of male workers from those with a significant presence of female workers. In some cases, an activity displays a prevalence of male workers together with a significant presence of female workers and is reported in both columns.

Male Workers are Preponderant or Almost Exclusive in the Workforce	Female Workers are a Significant Part of the Workforce (Preponderant in Some Cases)
Farmers	Farmers
Forestry and green areas maintenance workers	
Construction workers	
Fishing and shipping activities	
Beach activities	Beach activities
Offshore activities	
Asphalt workers	
Open sky miners	
Power line and water pipes workers	
Sky instructors and other outdoor winter workers	Sky instructors and other outdoor winter workers
Outdoor sport instructors	Outdoor sport instructors
Outdoor security activities (patrolling etc.)	Outdoor security activities (patrolling etc.)
Drivers (trucks, public transport etc.)	
Fuel station workers	
Mail carriers	Mail carriers
Outdoor loading and unloading activities	
Street vendors	Street vendors
	Kindergarten teachers
	School teachers

## Abbreviations

IRA	InfraRed A
SS	Solar Spectrum
SR	Solar Radiation
UVR	UltraViolet Radiation
ROS	Reactive Oxygen Species
UVA	UltraViolet A
UVB	UltraViolet B
IR	InfraRed
UVC	UltraViolet C
IRB	InfraRed B
IRC	InfraRed C
CCO	Cytochrome C Oxidase
Hsp	Heat-shock proteins
NO	Nitric Oxide
NF-kB	Nuclear Factor kappa-light-chain-enhancer of activated B cell
W/m^2^	Watt per square meter
J/m^2^	Joule per square meter
TPVR-1	Transient Potential Vanilloid Receptor 1
HRE	Hsp Responsive Elements
AP-1	Activator Protein 1
MMP-1	Matrix MetalloProteinase-1
J/cm^2^	Joule per square centimeter
mW/cm^2^	milliWatt per square centimeter
CC	Climate Change
DMBA	Dimethylbenz(a)anthracene
PAH	Polycyclic Aromatic Hydrocarbons

## References

[1] World Health Organization (2002). Global Solar UV Index: A Practical Guide: A Joint Recommendation of WHO, WMO, UNEP and ICNIRP.

[2] International Agency for Research on Cancer (IARC) (2012). A Review of Human Carcinogens, IARC Monographs on the Evaluation of Carcinogenic Risks to Humans, Vol. 100 D-Radiation.

[3] Kochevar I.E., Pathak M.A., Parrish J.A., Fitzpatrick (1999). Photophysics, photochemistry and photobiology. Dermatology in General Medicine.

[4] ICNIRP (International Commission on Non-Ionizing Radiation Protection) (2010). ICNIRP guidelines on limits of exposure to incoherent visible and infrared radiation. Health Phys..

[5] Sliney D. (2016). What is light? The visible spectrum and beyond. Eye.

[6] Barolet D., Christiaens F., Hamblin M.R. (2016). Infrared and skin: Friend or foe. J. Photochem. Photobiol. B Biol..

[7] EU-OSHA (European Agency for Safety and Health at Work) Outlook 1—New and Emerging Risks in Occupational Safety and Health. https://osha.europa.eu/en/publications/new-and-emerging-risks-occupational-safety-and-health/view.

[8] Turner S., Forman S.D., McNamee R., Wilkinson S.M., Agius R. (2015). Investigating work-related neoplasia associated with solar radiation. Occup. Med..

[9] Juzeniene A., Moan J. (2012). Beneficial effects of UV radiation other than via vitamin D production. Dermatoendocrinol.

[10] González Maglio D.H., Paz M.L., Leoni J. (2016). Sunlight effects on immune system: Is there something else in addition to UV-induced immunosuppression?. Biomed Res. Int..

[11] Grandi C., Borra M., Militello A., Polichetti A. (2016). Impact of climate change on occupational exposure to solar radiation. Ann. Ist. Super. Sanità.

[12] Modenese A., Korpinen L., Gobba F. (2018). Solar radiation exposure and outdoor work: An underestimated occupational risk. Int. J. Environ. Res. Public Health.

[13] Pludowski P., Grant W.B., Konstantynowicz J., Holick M.F. (2019). Editorial: Classic and pleiotropic actions of Vitamin D. Front. Endocriniol..

[14] Harms P.W. (2017). Update on Merkel cell carcinoma. Clin. Lab. Med..

[15] Lo Piccolo M.C., Lim H.W. (2010). Vitamin D in health and disease. Photodermatol. Photoimmunol. Photomed..

[16] Zhang R., Naughton D.P. (2010). Vitamin D in health and disease: Current perspectives. Nutr. J..

[17] Saraff V., Shaw N. (2016). Sunshine and vitamin D. Arch. Dis. Child..

[18] Ombra M.N., Paliogiannis P., Doneddu V., Sini M.C., Colombino M., Rozzo C., Stanganelli I., Tanda F., Cossu A., Palmieri G. (2017). Vitamin D status and risk for malignant cutaneous melanoma: Recent advances. Eur. J. Cancer Prev..

[19] Sowah D., Fan X., Dennett L., Hagtvedt R., Straube S. (2017). Vitamin D levels and deficiency with different occupations: A systematic review. BMC Public Health..

[20] Neale R.E., Khan S.R., Lucas R.M., Waterhouse M., Whiteman D.C., Olsen C.M. (2019). The effect of sunscreen on vitamin D: A review. Br. J. Dermatol..

[21] Hoel D.G., Berwick M., de Gruijl F.R., Holick M.F. (2016). The risks and benefits of sun exposure 2016. Dermatoendocrinol.

[22] Van der Rhee H.J., de Vries E., Coebergh J.W. (2016). Regular sun exposure benefits health. Med. Hypoth..

[23] Weller R.B. (2016). Sunlight has cardiovascular benefits independently of vitamin D. Blood Purif..

[24] Schieke S.M., Schroeder P., Krutmann J. (2003). Cutaneous effects of infrared radiation: From clinical observations to molecular response mechanisms. Photodermatol. Photoimmunol. Photomed..

[25] ICNIRP (International Commission on Non-Ionizing Radiation Protection) (2006). ICNIRP statement on far infrared radiation exposure. Health Phys..

[26] Sienkiewicz Z., van Rongen E., Croft R., Ziegelberger G., Veyret B. (2016). A closer look at the thresholds of thermal damage: Workshop report byan ICNIRP task group. Health Phys..

[27] Kleesz P., Darlenski R., Fluhr J.W. (2012). Full-body skin mapping for six biophysicalparameters: Baseline values at 16 anatomical sites in 125 human subjects. Skin Pharmacol. Physiol..

[28] Shan Q., Ma F., Wei J., Li H., Ma H., Sun P. (2019). Physiological functions of heat shock proteins. Curr. Protein Pept. Sci..

[29] Kettelhut E.A., Traylor J., Roach J.P. Erythema Ab Igne; Bookshelf, 2019 Bookshelf ID: NBK538250PMID:30855838. https://www.ncbi.nlm.nih.gov/books/NBK538250/.

[30] Ozturk M., An I. (2019). Clinical features and etiology of patients with erythema ab igne: A retrospective multicenter study. J. Cosmet. Dermatol..

[31] Akhalaya M., Maksimov G.V., Rubin A.B., Lademann J., Darvin M.E. (2014). Molecular action mechanisms of solar infrared radiation and heat on human skin. Ageing Res. Rew..

[32] Tsai S.-R., Hamblin M.R. (2017). Biological effects and medical applications of infrared radiation. J. Photochem. Photobiol. B Biol..

[33] Hamblin M.R. (2017). Mechanisms and applications of the anti-inflammatory effects of photobiomodulation. AIMS Biophys..

[34] Yadav A., Gupta A. (2017). Noninvasive red and near-infrared wavelength-induced photobiomodulation: Promoting impaired cutaneous wound healing. Photodermatol. Photoimmunol. Photomed..

[35] Karu T.I. (2008). Mitochondrial signalling in mammalian cells activated by red and near-IR radiation. Photochem. Photobiol..

[36] Migliario M., Sabbatini M., Mortellaro C., Renò F. (2018). Near infrared low-level laser therapy and cell proliferation: The emerging role of redox sensitive signal transduction pathways. J. Biophotonics.

[37] Sommer A.P. (2019). Mitochondrial cytochrome c oxidase is not the primary acceptor for near infrared light-it is mitochondrial bound water: The principles of low-level light therapy. Ann. Transl. Med..

[38] Schroeder P., Haendeler J., Krutmann J. (2008). The role of near infrared radiation in photoaging of the skin. Exp. Gerontol..

[39] Calles C., Schneider M., Macaluso F., Benesova T., Krutmann J., Schroeder P. (2010). Infrared A radiation influences the skin fibroblast transcriptome: Mechanisms and consequences. J. Investig. Dermatol..

[40] Sedlic F., Kovac Z. (2017). Non-linear actions of physiological agents: Finite disarrangements elicit fitness benefits. Redox Biol..

[41] Stone J., Mitrofanis J., Johnstone D.M., Falsini B., Bisti S., Adam P., Bravo Nuevo A., George-Weinstein M., Mason R., Eells J. (2018). Acquired resilience: An evolved system of tissue protection in mammals. Dose Response.

[42] Agathokleous E., Calabrese E.J. (2019). Hormesis: The dose response for the 21st century: The future has arrived. Toxicology.

[43] Barolet D. (2008). Light-emitting diodes (LEDs) in dermatology. Semin. Cutan. Med. Surg..

[44] Huang Y.Y., Sharma S.K., Carroll J., Hamblin M.R. (2011). Biphasic dose response in low level light therapy—An update. Dose Response.

[45] Mahmoud B.H., Hexsel C.L., Hamzavi I.H., Lim H.W. (2008). Effects of visible light on the skin. Photochem. Photobiol..

[46] Liebel F., Kaur S., Ruvolo E., Kollias N., Southall M.D. (2012). Irradiation of skin with visible light induces reactive oxygen species and matrix-degrading enzymes. J. Investig. Dermatol..

[47] Cadet J., Douki T., Ravanat J.-L. (2015). Oxidatively generated damage to cellular DNA by UVB and UVA radiation. Photochem. Photobiol..

[48] Vandersee S., Beyer M., Lademann J., Darvin M.E. (2015). Blue-violet light irradiation dose dependently decreases carotenoids in human skin, which indicates the generation of free radicals. Oxid. Med. Cell. Longev..

[49] Passaglia Schuch A., Cestari Moreno N., Schuch N.J., Martins Menck C.F., Carrião Machado Garcia C. (2017). Sunlight damage to cellular DNA: Focus on oxidatively generated lesions. Free Rad. Biol. Med..

[50] Nakashima Y., Ohta S., Wolf A.M. (2017). Blue light-induced oxidative stress in live skin. Free Rad. Biol. Med..

[51] Lushchak V.I. (2014). Free radicals, reactive oxygen species, oxidative stress and its classification. Chem. Biol. Interact..

[52] Rahal A., Kumar A., Singh V., Yadav B., Tiwari R., Chakraborty S., Dhama K. (2014). Oxidative stress, prooxidants, and antioxidants: The interplay. BioMed Res. Int..

[53] Kohli I., Chaowattanapanit S., Mohammad T.F., Nicholson C.L., Fatima S., Jacobsen G., Kollias N., Lim H.W., Hamzavi I.H. (2018). Synergistic effects of long-wavelength ultraviolet A1 and visible light on pigmentation and erythema. Br. J. Dermatol..

[54] Calapre L., Gray E.S., Ziman M. (2013). Heat stress: A risk factor for skin carcinogenesis. Cancer Lett..

[55] Calapre L., Gray E.S., Kurdykowski S., David A., Hart P., Descargues P., Mel Ziman M. (2016). Heat-mediated reduction of apoptosis in UVB-damaged keratinocytes in vitro and in human skin ex vivo. BMC Dermatol..

[56] Sottile M.L., Silvina B., Nadin S.B. (2018). Heat shock proteins and DNA repair mechanisms: An updated overview. Cell Stress Chaperones.

[57] Petersen B., Philipsen P.A., Wulf H.C. (2014). Skin temperature during sunbathing–relevance for skin cancer. Photochem. Photobiol. Sci..

[58] Bain J.A., Rusch H.P. (1943). Carcinogenesis with ultraviolet radiation of wavelength 2800–3400 A. Cancer Res..

[59] Bain J.A., Rusch H.P., Line B.E. (1943). The effect of temperature upon ultraviolet carcinogenesis with wavelength 2800–3400 A. Cancer Res..

[60] Freeman R.G., Knox J.M. (1964). Influence of temperature on ultraviolet injury. Arch. Dermatol..

[61] Van der Leun J.C., de Gruijl F.R. (2002). Climate change and skin cancer. Photochem. Photobiol. Sci..

[62] Van der Leun J.C., Piacentini R.D., de Gruijl F.R. (2008). Climate change and human skin cancer. Photochem. Photobiol. Sci..

[63] Lan C.E. (2019). Effects and interactions of increased environmental temperature and UV radiation on photoageing and photocarcinogenesis of the skin. Exp. Dermatol..

[64] IPCC (Intergovernmental Panel on Climate Change) (2014). AR5 Synthesis Report: Climate Change 2014. https://www.ipcc.ch/report/ar5/syr/.

[65] Bais A.F., McKenzie R.L., Bernhard G., Aucamp P.J., Ilyas M., Madronich S., Tourpali K. (2015). Ozone depletion and climate change: Impacts on UV radiation. Photochem. Photobiol. Sci..

[66] Damian D.L., Matthews Y.J., Phan T.A., Halliday G.M. (2011). An action spectrum for ultraviolet radiation-induced immunosuppression in humans. Br. J. Dermatol..

[67] Halliday G.M., Byrne S.N., Damian D.L. (2011). Ultraviolet A radiation: Its role in immunosuppression and carcinogenesis. Semin. Cutan. Med. Surg..

[68] Gibbs N.K., Norval M. (2013). Photoimmunosuppression: A brief overview. Photodermatol Photoimmunol Photomed..

[69] Matos T.R., Sheth V. (2016). The symbiosis of phototherapy and photoimmunology. Clin. Dermatol..

[70] Grandi C., D’Ovidio M.C. (2018). The interplay between solar radiation, climate change and immunotoxicants in relation to immune response modulation: A Concern for outdoor workers’ health. Am. J. Health Res..

[71] Grandi C., D’Ovidio M.C. (2019). Outdoor Workers Exposed to Solar Radiation: A Spotlight on Immunomodulation. Biomed. J. Sci. Tech. Res..

[72] Norval M. (2006). The effect of ultraviolet radiation on human viral infections. Photochem. Photobiol..

[73] Guo B., Naish S., Hu W., Tong S. (2015). The potential impact of climate change and ultraviolet radiation on vaccine preventable infectious diseases and immunization service delivery system. Exp. Rev. Vaccines.

[74] Swaminathan A., Harrison S.L., Ketheesan N., van den Boogaard C.H.A., Dear K., Allen M., Hart P.H., Cook M., Lucas R.M. (2019). Exposure to solar UVR suppresses cell-mediated immunization responses in humans: The Australian ultraviolet radiation and immunity study. J. Investig. Dermatol..

[75] Patra V.K., Scott N., Byrne S.N., Wolf P. (2016). The skin microbiome: Is it affected by UV-induced immune suppression?. Front. Microbiol..

[76] Didona D., Paolino G., Bottoni U., Cantisani C. (2018). Non melanoma skin cancer pathogenesis overview. Biomedicines.

[77] Hart P.H., Norval M. (2018). Ultraviolet radiation-induced immunosuppression and its relevance for skin carcinogenesis. Photochem. Photobiol. Sci..

[78] Collins L., Quinn A., Stasko T. (2019). Skin cancer and immunosuppression. Dermatol. Clin..

[79] Beachy S.H., Repasky E.A. (2011). Toward establishment of temperature thresholds for immunological impact of heat exposure in humans. Int. J. Hypertherm..

[80] Zininga T., Ramatsui L., Shonhai A. (2018). Heat shock proteins as immunomodulants. Molecules.

[81] Yusuf N., Nasti T.H., Ahmad I., Chowdhury S., Mohiuddin H., Xu H., Athar M., Timares L., Craig A., Elmets C.A. (2015). In vivo suppression of HSP27 and HSP70 accelerates DMBA-induced skin carcinogenesis by inducing antigenic unresponsiveness to the initiating carcinogenic chemical. J. Immunol..

[82] Lee C.-H., Hong C.-H., Liaod W.-T., Yu H.-S. (2016). Differential immunological effects of infrared irradiation and its associated heat in vivo. J. Photochem. Photobiol. B Biol..

[83] Xiang J., Bi P., Pisaniello D., Hansen A. (2014). Health impacts of workplace heat exposure: An epidemiological review. Ind. Health.

[84] D’Ovidio M.C., Grandi C., Marchetti E., Polichetti A., Iavicoli S. (2016). Climate change and occupational health. Ann. Ist. Super. Sanità.

[85] Moda H.M., Filho W.L., Minhas A. (2019). Impacts of climate change on outdoor workers and their safety: Some research priorities. Int. J. Environ. Res. Public Health.

[86] Applebaum K.M., Graham J., Gray G.M., LaPuma P., McCormick S.A., Northcross A., Perry M.J. (2016). An overview of occupational risks from climate change. Curr. Environ. Health Rep..

[87] Levi M., Kjellstrom T., Baldasseroni A. (2018). Impact of climate change on occupational health and productivity: A systematic literature review focusing on workplace heat. Med. Lav..

[88] Modenese A., Ruggieri F.P., Bisegna F., Borra M., Burattini C., Della Vecchia E., Grandi C., Grasso A., Gugliermetti L., Manini M. (2019). Occupational exposure to solar UV radiation of a group of fishermen working in the Italian North Adriatic sea. Int. J. Environ. Res. Public Health.

[89] (1989). Council Directive 89/391/EEC of 12 June 1989 on the introduction of measures to encourage improvements in the safety and health of workers at work. Off. J. Eur. Communities.

[90] Gies P., van Deventer E., Green A.C., Sinclair C., Tinker R. (2018). Review of the global solar UV index 2015 workshop report. Health Phys..

[91] Godar D.E. (2005). UV doses worldwide. Photochem. Photobiol..

[92] Modenese M., Bisegna F., Borra M., Grandi C., Gugliermetti F., Militello A., Gobba F. (2016). Outdoor work and solar radiation exposure: Evaluation method for epidemiological studies. Med. Pracy.

[93] Wittlich M., Westerhausen S., Kleinespel P., Rifer G., Stoppelmann W. (2016). An approximation of occupational lifetime UVR exposure: Algorithm for retrospective assessment and currentmeasurements. JEADV.

[94] Vecchia P., Hietanen M., Stuck B.E., van Deventer E., Niu S., ICNIRP (International Commission on Non-Ionizing Radiation Protection), ILO (International Labour Organization), WHO (World Health Organization) (2007). Protecting Workers from Ultraviolet Radiation.

[95] Schalka S., Steiner D., Ravelli F.N., Steiner T., Terena A.C., Marçon C.R., Ayres E.L., Addor F.A., Miot H.A., Ponzio H. (2014). Brazilian consensus on photoprotection. An. Bras. Dermatol..

[96] European Dermatology Forum European Dermatology Guideline for the photodermatoses. 3. Photoprotection. https://www.edf.one/dam/jcr:46edc745-1bdd-459c-a323-b7e48971891f/guideline_Photoprotection.pdf.

[97] Kramer D.M., Tenkate T., Strahlendorf P., Kushner R., Gardner A., Holness D.L. (2015). Sun Safety at Work Canada: A multiple case-study protocol to develop sun safety and heat protection programs and policies for outdoor workers. Implement. Sci..

[98] Cestari T., Buster K. (2017). Photoprotection in specific populations: Children and people of color. J. Am. Acad. Dermatol..

[99] Whiteman D.C., Neale R.E., Aitken J., Gordon L., Green A.C., Janda M., Olsen C.M., Soyer H.P., on behalf of the Sunscreen Summit Policy Group (2019). When to apply sunscreen: A consensus statement for Australia and New Zealand. Aust. New Zealand J. Public Health..

[100] Cancer Council Victoria, Victorian Health Promotion Foundation (VicHealth) Sun Smart. https://www.sunsmart.com.au/.

[101] ICNIRP (International Commission on Non Ionizing Radiation Protection) (2010). Statement–protection of workers against ultraviolet radiation. Health Phys..

[102] Reinau D., Weiss M., Meier C.R., Diepgen T.L., Surber C. (2013). Outdoor workers’ sun-related knowledge, attitudes and protective behaviours: A systematic review of cross-sectional and interventional studies. Br. J. Dermatol..

[103] Rocholl M., Ludewig M., John S.M., Bitzer E.M., Wilke A. (2019). Outdoor workers’ perceptions of skin cancer risk and attitudes to sun-protective measures: A qualitative study. J. Occup. Health.

[104] Weber M., Uller A., Schulmeister K., Brusl H., Hann H., Kindl P. (2007). Outdoor workers’ acceptance of personal protective measures against solar ultraviolet radiation. Photochem. Photobiol..

[105] Meinke M.C., Haag S.F., Schanzer S., Groth N., Gersonde I., Lademann J. (2011). Radical protection by sunscreens in the infrared spectral range. Photochem. Photobiol..

[106] Grether-Beck S., Marini A., Thomas Jaenicke T., Krutmann J. (2015). Effective photoprotection of human skin against infrared A radiation by topically applied antioxidants: Results from a vehicle controlled, double-blind, randomized study. Photochem. Photobiol..

[107] Dupont E., Gomez J., Bilodeau D. (2013). Beyond UV radiation: A skin under challenge. Int. J. Cosmetic Sci..

[108] Sondenheimer K., Krutmann J. (2018). Novel means for photoprotection. Front. Med..

[109] Stengel F. (2018). Homeostasis in topical photoprotection: Getting the spectral balance right. Am. J. Clin. Dermatol..

[110] (2006). Directive 2006/25/EC of the European Parliament and of the Council of 5 April 2006 on the minimum health and safety requirements regarding the exposure of workers to risks arising from physical agents (artificial optical radiation) (19th individual Directive within the meaning of Article 16(1) of Directive 89/391/EEC). Off. J. Eur. Union.

[111] Grandi C., Militello A., Borra M. (2018). The role of near infrared with regard to potential long-term adverse effects in outdoor workers exposed to solar radiation. Occup. Environ. Med..

[112] Brand R.M., Wipf P., Durham A., Michael W., Epperly M.W., Greenberger J.S., Falo L.D. (2018). Targeting mitochondrial oxidative stress to mitigate UV-induced skin damage. Front. Pharmacol..

[113] Mendelsohn A.R., Larrick J.W. (2014). Paradoxical effects of antioxidants on cancer. Rejuvenation Res..

[114] Pisoschi A.M., Pop A. (2015). The role of antioxidants in the chemistry of oxidative stress: A review. Eur. J. Med. Chem..

[115] Da Silva M.B., da Silva Melo A.R., de Araújo Costa L., Barroso H., de Oliveira N.F.P. (2017). Global and gene-specific DNA methylation and hydroxymethylation in human skin exposed and not exposed to sun radiation. An. Bras. Dermatol..

[116] Wild C.P. (2005). Complementing the genome with an “exposome”: The outstanding challenge of environmental exposure measurement in molecular epidemiology. Cancer Epidemiol. Biomark. Prev..

[117] Siroux V., Agier L., Slama R. (2016). The exposome concept: A challenge and a potential driver for environmental health research. Eur. Respir. Rev..

[118] Holland N. (2017). Future of environmental research in the age of epigenomics and exposomics. Rev. Environ. Health.

